# Relationships Between Salivary Biomarkers and Oral Function in Elderly People Using a Saliva Testing Device

**DOI:** 10.3390/dj14050251

**Published:** 2026-04-24

**Authors:** Toshiro Yamamoto, Keiichi Ishizaki, Yukina Muraoka, Ibuki Ishikaku, Yusuke Tomiie, Hideki Yoshimatsu, Keita Kano, Norihiro Ouchi, Atsuo Adachi, Satoaki Matoba

**Affiliations:** 1 Department of Dental Medicine, Graduate School of Medical Science, Kyoto Prefectural University of Medicine, Kyoto 602-8566, Japan; ik0725@koto.kpu-m.ac.jp (K.I.); yt1207@koto.kpu-m.ac.jp (Y.M.); ishikaku@koto.kpu-m.ac.jp (I.I.); yusuke@koto.kpu-m.ac.jp (Y.T.); yoshimat@koto.kpu-m.ac.jp (H.Y.); january301983@gmail.com (K.K.); 2 Department of Longevity and Community Health and Medicine, Graduate School of Medical Science, Kyoto Prefectural University of Medicine, Kyoto 602-8566, Japan; oouchino@koto.kpu-m.ac.jp (N.O.); adachia@koto.kpu-m.ac.jp (A.A.); 3 Kyotango City Yasaka Hospital, Kyotango 627-0111, Japan; 4 Department of Cardiovascular Medicine, Graduate School of Medical Science, Kyoto Prefectural University of Medicine, Kyoto 602-8566, Japan; matoba@koto.kpu-m.ac.jp

**Keywords:** salivary biomarkers, oral function, oral functional decline (oral hypofunction), elderly people, saliva testing device, masticatory function, tongue–lip motor function

## Abstract

**Background/Objectives**: Oral functional decline is associated not only with impaired oral conditions, such as poor oral hygiene, oral dryness, decreased occlusal force, reduced masticatory performance, low tongue pressure, impaired tongue–lip motor function, and dysphagia, but also with deterioration in overall systemic health. Saliva plays a crucial role in maintaining oral function. This study investigated the relationships between conditions related to oral functional decline and salivary biomarkers. **Methods**: Elderly people residing in the Kyotango area were evaluated using oral function assessments, including the oral hygiene status, salivary flow rate, occlusal force, masticatory performance, tongue pressure, tongue–lip motor function, and swallowing function. Salivary biomarkers were evaluated using an oral environment assessment system (SillHa, Arkray, Kyoto). The biomarkers tested included cariogenic bacteria, acidity, buffering capacity, leukocytes, protein, and ammonia. Statistical analyses were performed using SPSS version 30 (IBM, Armonk, NY, USA). **Results**: Significant differences and correlations were observed between salivary biomarkers and five of the seven assessment criteria for oral functional decline: a poor oral hygiene status, oral dryness, decreased occlusal force, impaired tongue–lip motor function, and decreased masticatory function. A poor oral hygiene status was associated with the bacterial count and cariogenic bacteria. Oral dryness was associated with the salivary flow rate, acidity, and buffering capacity. Decreased occlusal force was associated with the number of remaining teeth, cariogenic bacteria, acidity, leukocytes, and protein. Impaired tongue–lip motor function was associated with the /pa/, /ta/, and /ka/ sounds and leukocytes. Decreased masticatory function was associated with masticatory performance, cariogenic bacteria, leukocytes, and protein. **Conclusions**: The present results suggest the potential of salivary biomarker testing using this assessment system as a simple and practical screening tool for identifying oral functional decline. Furthermore, salivary biomarker testing may be useful in oral function assessments.

## 1. Introduction

In elderly people, a deterioration in the oral hygiene status, regarded as oral functional decline, may interfere with the basic activities of daily living and contribute to the development of frailty [1]. Frailty is characterized by age-related declines in physical and mental functions, represents an intermediate stage between a healthy state and the need for long-term care, and has been associated with decreased oral motor ability as well as impairments in mastication, swallowing, and salivary secretion [2]. Oral functional decline is associated with worsening systemic health [3], and must not be regarded as a dental issue in an aging society due to its effects on overall health and quality of life, including nutrition, muscle strength, cognition, and social participation. Saliva may be collected non-invasively and easily and contains a wide range of biological information, including hormonal, metabolic, immunological, and microbial components. Due to these characteristics, numerous studies have investigated the use of saliva as a health indicator in conditions such as dementia, cancer, diabetes, non-communicable diseases, and COVID-19 [4,5,6,7,8]. Research has been actively conducted on salivary biomarkers for Alzheimer’s disease, with salivary lactoferrin, amyloid-β, and tau attracting considerable attention [9]. Although salivary lactoferrin is affected by periodontal disease [10], saliva may also be associated with oral diseases other than inflammatory conditions, such as periodontitis. Salivary biomarkers, including calprotectin, 8-hydroxy-2′-deoxyguanosine, and advanced glycation end products, have been associated with oral functional decline in elderly people [11]. Furthermore, oral functional decline correlated with systemic health conditions, such as frailty and mild cognitive impairment, with decreased occlusal force and low tongue pressure being identified as significant risk factors for mild cognitive impairment [12]. An oral function assessment involves objectively evaluating whether an oral function is being performed normally or has declined. Oral hypofunction is defined as a condition in which oral function declines due to aging or disease and functional deterioration is detected in multiple assessments. Specifically, it is diagnosed when three or more of the following are impaired: the oral hygiene status, salivary flow rate, masticatory performance, occlusal force, tongue pressure, tongue–lip motor function, and swallowing function [13]. However, these assessments require multiple specialized devices and expert evaluations. The salivary biomarker tests employed in the present study have been suggested to complement treatment planning in routine clinical practice when used in conjunction with clinical examination and medical history [14]; however, it remains unclear whether these tests provide information that is beneficial for oral function assessment, particularly as a screening tool for oral functional decline.

Therefore, the present study examined the relationships between various salivary biomarkers used in a non-invasive and simple saliva test and oral function assessments, and investigated whether these salivary biomarkers serve as a screening tool or supportive method for identifying oral functional decline.

## 2. Materials and Methods

### 2.1. Participants

This was a cross-sectional analysis conducted as part of the Kyotango Multipurpose Cohort Study. Between January 2019 and December 2025, 294 community-dwelling residents aged ≥70 years living in the Kyotango area in northern Kyoto Prefecture, Japan were included as the elderly group and underwent salivary biomarker testing and oral function assessments. In 2010, Japan became a super-aging society, with more than 21% of its population aged ≥65 years. The Kyotango region has a population of less than 100,000, with more than 38% aged ≥65 years, representing the highest aging rate in Kyoto Prefecture. The region is located on a peninsula approximately 130 km north of an urban area (Kyoto city) and is surrounded by the sea and mountains. Due to its geographically disadvantaged conditions, including limited accessibility, the region has attracted attention as a longevity area, with more than 200 centenarians per 100,000 population despite population decline and aging. As a control group, 54 healthy adults in their 20 s and 30 s residing in an urban area (Kyoto city) were included and underwent salivary biomarker testing and oral function assessments. All subjects were volunteers, and the examinations were conducted in a hospital examination room. These were conducted before lunch, and teeth were not brushed before the evaluation.

### 2.2. Salivary Biomarker Testing

**Figure 1:**
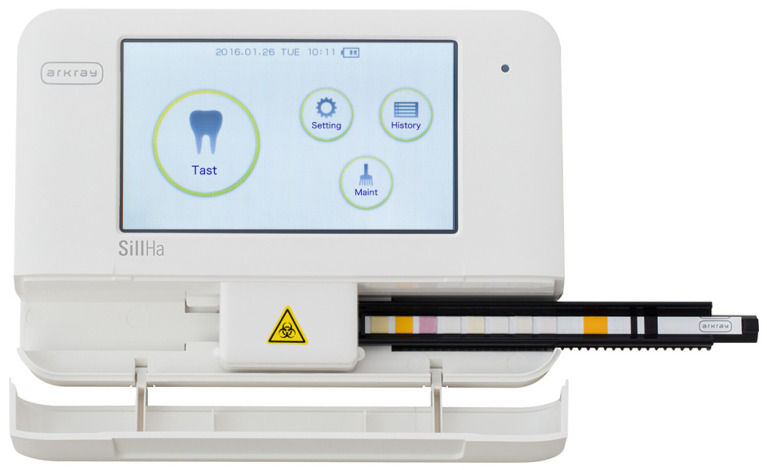
SillHa multipoint saliva analyzer.

Participants rinsed their mouths with 3 mL of sterile water for 10 s and then expectorated into a collection cup. The mouth-rinse expectorate obtained was used as the sample. The sample was dropped onto test strips of the oral environment assessment system (SillHa, Arkray, Kyoto, Japan), and color changes on the test strips were measured using an analyzer based on a dual-wavelength reflectance photometry method, with reflectance measured at wavelengths of 565, 635, and 760 nm (Figure 1). The parameters measured included cariogenic bacteria, acidity, buffering capacity, leukocytes, protein, and ammonia. Measurement results were displayed on a scale ranging from 0 to 100. The corresponding measurement ranges were as follows: cariogenic bacteria, 10^6^–10^8^ CFU/mL; acidity, pH 6.0–8.0; buffering capacity, pH 2.8–6.0; blood, 0–0.50 mg/dL; leukocytes, 0–200 U/L; protein, 0–60 mg/dL; and ammonia, 0–10,000 N-mg/dL [14]. To select evaluation thresholds, age-specific mean values were calculated for each parameter, and boundary values were defined as the mean ± 0.5 standard deviation. The evaluation was performed using results based on a population dataset of 87,451 individuals. This is based on data regarding saliva biomarker test results, age, and sex measured at dental clinics located throughout Japan that subscribed to the cloud-based free optional service between August 2023 and July 2025. This study was conducted with the approval of the Medical Ethics Committee of Kyoto Prefectural University of Medicine (approval number: ERB-C-885; approval date: 24 January 2018).

### 2.3. Oral Function Assessments

#### 2.3.1. Oral Hygiene Status

The surface of the tongue was swabbed 3 times back and forth using a sterile cotton swab, and the bacterial count was measured using a microbial quantitative analysis device (Oral Bacterial Counter, Panasonic, Tokyo, Japan). A bacterial count of ≥3.162 × 10^6^ CFU/mL was defined as a poor oral hygiene status.

#### 2.3.2. Salivary Flow Rate

The stimulated salivary flow rate was measured using the Saxon test. Participants chewed a piece of dry gauze for 2 min, after which the gauze containing saliva was collected as a single mass and weighed. The increase in weight was defined as the stimulated salivary flow rate. A value of ≤2 g/2 min was considered to be indicative of oral dryness.

#### 2.3.3. Occlusal Force

The number of remaining teeth was counted after excluding residual roots and teeth with mobility grade 3. A remaining tooth count of ≤19 was defined as decreased occlusal force.

#### 2.3.4. Tongue–Lip Motor Function

Participants were instructed to pronounce the syllables /pa/, /ta/, and /ka/ as quickly as possible for 5 s each, and the number of repetitions was recorded using an automatic counter (Kenkou-kun Handy, SANKA Co., Ltd., Niigata, Japan). Tongue–lip motor function was considered to be impaired if the number of repetitions per second was <6 for any of the 3 syllables.

#### 2.3.5. Masticatory Performance

Participants chewed a glucose-containing gummy candy for 20 s, after which the amount of eluted glucose was measured using a glucose sensor (Gluco Sensor GS-Ⅱ, GC Corporation, Tokyo, Japan). A glucose concentration of <100 mg/dL was defined as decreased masticatory function.

#### 2.3.6. Tongue Pressure

Tongue motor function was evaluated by measuring the maximum voluntary tongue pressure using a tongue pressure measurement device (Tongue Pressure Measurement Device, JMS Co., Ltd., Hiroshima, Japan). A tongue pressure of <30 kPa was defined as low tongue pressure.

#### 2.3.7. Swallowing Function

Swallowing function was assessed using the Eating Assessment Tool-10 (EAT-10), a 10-item questionnaire-based simple screening tool for dysphagia. A total score of ≥3 was defined as decreased swallowing function.

### 2.4. Statistical Analysis

Statistical analyses were performed using SPSS version 30 (IBM, Armonk, NY, USA). Relationships between salivary biomarkers and each oral function assessment were evaluated using non-parametric methods. Group comparisons were conducted using the Mann–Whitney U test, and relationships were analyzed using Spearman’s rank correlation coefficient. A *p*-value < 0.05 was considered to be significant. This study was conducted with the approval of the Medical Ethics Committee of Kyoto Prefectural University of Medicine (approval number: ERB-C-885).

## 3. Results

### 3.1. Details of Research Participants

**Figure 2:**
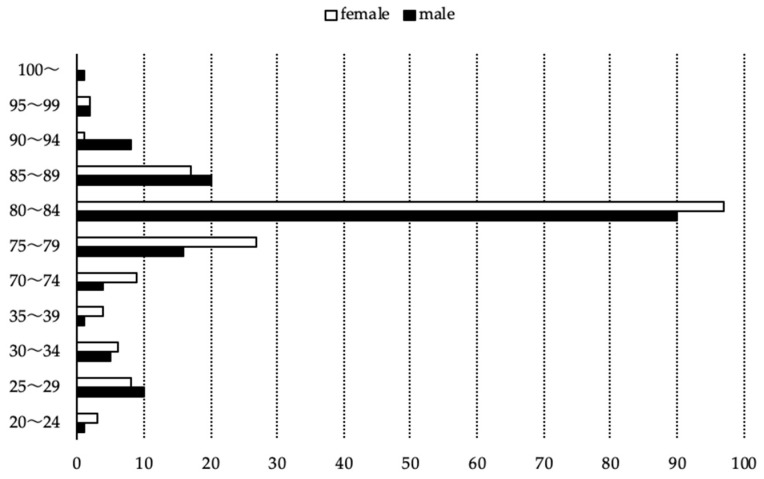
Sex and age distributions.

Among the 294 participants in the elderly group, 141 were men and 153 were women. Ages ranged from 71 to 101 years, with the 80–84-year age group being the largest. Among the 54 participants in the control group, 17 were men and 21 were women. Ages ranged from 21 to 37 years, with the 25–29-year age group being the largest (Figure 2).

### 3.2. Oral Function Assessments

All oral function assessments revealed significant differences between the elderly and control groups based on analyses using the Mann–Whitney U test (Table 1).

### 3.3. Salivary Biomarker Testing

Among the salivary biomarkers tested, all parameters, except for buffering capacity, showed significant differences between the elderly and control groups based on analyses using the Mann–Whitney U test (Table 2).

### 3.4. Analysis of the Oral Environment Based on Salivary Biomarker Testing

**Figure 3:**
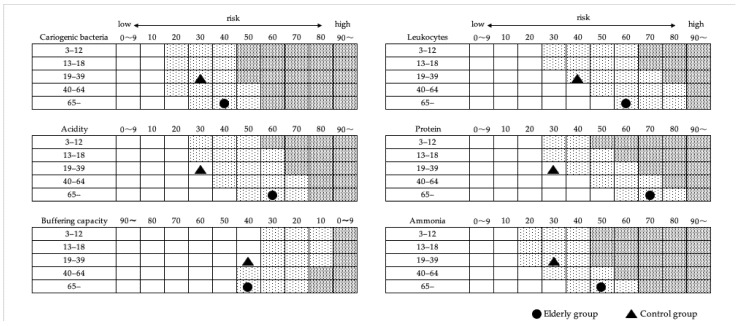
Comparison of salivary biomarker risk distributions across age groups in Kyotango and nationwide Japan. 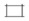 Low Risk, 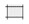 Medium Risk, 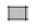 High Risk.

The mean values of salivary biomarkers measured in the saliva of the elderly group were at average levels relative to the risk level for each parameter calculated from national reference data (Figure 3). Among the salivary biomarkers tested, risk levels for cariogenic bacteria, leukocytes, acidity, protein, and ammonia were higher in the elderly group than in the control group. In contrast, buffering capacity was similar between the 2 groups.

### 3.5. Relationships Between Oral Function Assessments and Salivary Biomarker Testing in the Elderly Group

Significant differences were observed between oral function assessments and salivary biomarkers using the Mann–Whitney U test, specifically for the oral hygiene status and cariogenic bacteria; the salivary flow rate and acidity and buffering capacity; occlusal force and cariogenic bacteria, acidity, leukocytes, and protein; tongue–lip motor function and leukocytes; and masticatory performance and cariogenic bacteria, leukocytes, and protein. In addition, these parameters showed correlations based on Spearman’s rank correlation coefficient analysis. Correlations were observed for masticatory performance and leukocytes (Table 3). These results indicate that salivary biomarkers were associated with a poor oral hygiene status, oral dryness, decreased occlusal force, reduced masticatory performance, and decreased tongue–lip motor function, which are components used to evaluate oral functional decline (Table 4).

### 3.6. Relationships of the Salivary Flow Rate with Salivary Biomarkers and Oral Function Assessments in the Elderly Group

The salivary flow rate correlated with salivary biomarkers (Table 3). Furthermore, it showed significant differences in relation to occlusal force and masticatory performance based on the Mann–Whitney U test and correlations in Spearman’s rank correlation coefficient analysis (Table 5).

## 4. Discussion

Previous studies indicated the potential of salivary biomarkers for the diagnosis and monitoring of periodontal disease and peri-implantitis [15,16]. More recently, salivary biomarker testing has been reported not only to serve as a biomarker for periodontal treatment [17,18,19], but also to be helpful in treatment planning [14]. In addition, such testing was shown to be useful for both an oral health risk assessment and as an early screening tool for the cognitive status [20] and sleep disorders [21]. However, evidence for the relationships between comprehensive salivary biomarker profiles and clinically defined oral functional decline in a very elderly population remains limited. Against this background, the present study examined the utility of salivary biomarker testing as a screening tool for oral functional decline. Since dental caries is a multifactorial disease, dental health was assessed using 3 parameters: cariogenic bacteria, acidity, and buffering capacity. Gingival health was evaluated using leukocytes and protein levels, while oral cleanliness was assessed using ammonia as an indicator, thereby enabling a comprehensive evaluation of the oral environment.

The present study investigated elderly people living in a longevity region in Japan. An assessment of the oral environment in this group revealed that the mean values of each salivary biomarker were similar to the risk levels calculated from national average data in Japan. However, overall risk levels were generally higher in the elderly group than in the younger control group, indicating a poorer oral hygiene status (Figure 2). In addition, salivary biomarker levels were significantly lower in the elderly group than in the control group for all parameters, except for buffering capacity (Table 2). Since salivary biomarker levels in elderly people vary more than those in younger individuals [22], further comparative analyses with salivary biomarker data from other regions are planned. It is important to note that the mean values used in the present study represent indicators for a relative evaluation rather than absolute reference values (normal ranges). This limitation may be partly attributable to salivary components being highly susceptible to variations caused by the time of day, dietary intake, and stress [23,24].

A condition under which multiple aspects of oral function decline due to combined factors is referred to as oral hypofunction, which differs from conventional structural impairments, such as dental caries or tooth loss. The diagnosis of oral hypofunction requires an assessment of the 7 items tested in this study: the oral hygiene status, salivary flow rate, occlusal force, masticatory performance, tongue pressure, tongue–lip motor function, and swallowing function. However, it is often difficult to rapidly perform these assessments in clinical practice because they require the preparation and maintenance of multiple devices, making the process complex and burdensome. The elderly group showed significantly impaired functions in all assessments from those in the control group (Table 1). In the present study, 5 of the 7 items, namely the oral hygiene status, salivary flow rate, masticatory performance, tongue–lip motor function, and occlusal force, showed significant differences and correlations with salivary biomarkers (Table 3 and Table 4). While it is self-evident that a poor oral hygiene status and oral dryness are affected by saliva, saliva has also been implicated in decreases in occlusal force [25], masticatory performance [26], and tongue–lip motor function [27]. In the present study, the salivary flow rate correlated with occlusal force and masticatory performance (Table 5). These results suggest that reduced salivary flow was associated not only with the direct effects of aging on the salivary glands, but also decreased oral muscle strength. Tongue–lip motor function was expected to have an impact on salivary flow through the stimulation of the salivary glands; however, a correlation was not observed between the salivary flow rate and tongue–lip motor function in the present study. Therefore, further investigations with a larger sample size are warranted.

Saliva testing is easier, cheaper, and less invasive than blood sampling. If simple screening using saliva testing is established for elderly people and in community healthcare settings, it may contribute to low-burden health management and early detection. In other words, managing oral function in addition to oral hygiene (including the assessment of dental caries and periodontal disease) may help prevent the exacerbation of dental diseases and contribute to the maintenance of and improvements in infection control and the nutritional status in the elderly.

Therefore, these results suggest the potential of salivary biomarker testing using the oral environment assessment system as a screening tool for oral hypofunction.

## 5. Limitations

Further investigations linking the present results with medical data are required to clarify the relationship with overall systemic health. However, the majority of studies conducted to date have been cross-sectional, and a causal relationship between oral functional decline and health deterioration has yet to be sufficiently established. Saliva is affected by a number of factors, such as food intake, smoking, and toothbrushing, and several challenges remain for its clinical application, including the lack of standardization in sampling procedures (e.g., collection methods and timing, storage conditions, and measurement techniques), variability due to demographic factors and oral conditions, and issues related to reproducibility. Furthermore, since no direct evaluation of frailty was conducted, it was not possible to assess the relationship between frailty and aging. Therefore, although salivary biomarkers may represent “promising indicators of future health”, they are currently regarded as “supplementary and exploratory”.

## 6. Conclusions

In elderly people living in a longevity region, the results of salivary biomarker testing correlated with those obtained in evaluations of the oral hygiene status, salivary flow rate, occlusal force, masticatory performance, and tongue–lip motor function, which are key indicators of oral function. Based on these results, salivary biomarker testing may be useful in oral function assessments.

## Figures and Tables

**Table 1:** Oral function assessments in elderly and control groups.

		Elderly	Control	*p*-Value
Oral hygiene status		2.4 ± 6.2	2.9 ± 2.6	*p* < 0.01
Salivary flow rate		4.5 ± 3.1	5.9 ± 2.2	*p* < 0.01
Occlusal force		630.2 ± 435.6	1056.3 ± 550.4	*p* < 0.01
Tongue–lip motor function	/pa/	5.7 ± 1.1	6.6 ± 0.8	*p* < 0.01
	/ta/	5.6 ± 1.0	7.0 ± 1.3	*p* < 0.01
	/ka/	5.3 ± 1.0	6.4 ± 1.2	*p* < 0.01
Masticatory performance		160.1 ± 79.5	224.6 ± 78.1	*p* < 0.01
Tongue pressure		29.5 ± 7.6	41.2 ± 9.2	*p* < 0.01
Swallowing function		0.9 ± 2.2	0.0 ± 0.1	*p* < 0.01

The Mann–Whitney U test, SPSS 30.

**Table 2:** Salivary biomarker testing in elderly and control groups.

	Elderly	Control	*p*-Value
Cariogenic bacteria	47.2 ± 29.9	30.4 ± 14.8	*p* < 0.01
Acidity	61.2 ± 26.5	39.2 ± 23.9	*p* < 0.01
Buffering capacity	43.5 ± 25.3	42.0 ± 30.2	N.S.
Leukocytes	65.8 ± 31.4	40.6 ± 26.5	*p* < 0.01
Protein	70.1 ± 24.6	36.8 ± 15.8	*p* < 0.01
Ammonia	56.8 ± 49.0	38.0 ± 20.5	*p* < 0.01

N.S.: Not Significant. The Mann–Whitney U test, SPSS 30.

**Table 3:** Relationships between salivary biomarkers and markers of oral functional decline.

	1. Oral Hygiene Status	2. Salivary Flow Rate	3. Occlusal Force
Cariogenic bacteria	*p* < 0.01 r = −0.175, *p* < 0.01	N.S.	*p* < 0.05 r = 0.126, *p* < 0.05
Acidity	N.S.	*p* < 0.01 r = −0.119, *p* < 0.01	*p* < 0.05 r = −0.132, *p* < 0.05
Buffering capacity	N.S.	*p* < 0.01 r = 0.212, *p* < 0.01	N.S.
Leukocytes	N.S.	N.S.	*p* < 0.01 r = 0.267, *p* < 0.01
Protein	N.S.	N.S.	*p* < 0.05 r = 0.165, *p* < 0.05
Ammonia	N.S.	N.S.	N.S.
	4. Tongue–lip motor function		
	/pa/	/ta/	/ka/
Cariogenic bacteria	N.S.	N.S.	N.S.
Acidity	N.S.	N.S.	N.S.
Buffering capacity	N.S.	N.S.	N.S.
Leukocytes	*p* < 0.01 r = 0.213, *p* < 0.01	*p* < 0.01 r = 0.137, *p* < 0.01	*p* < 0.01 r = 0.125, *p* < 0.05
Protein	N.S.	N.S.	N.S.
Ammonia	N.S.	N.S.	N.S.
	5. Masticatory performance	6. Tongue pressure	7. Swallowing function
Cariogenic bacteria	*p* < 0.05 r = 0.166, *p* < 0.01	N.S.	N.S.
Acidity	N.S.	N.S.	N.S.
Buffering capacity	N.S.	N.S.	N.S.
Leukocytes	*p* < 0.01 r = 0.277, *p* < 0.01	N.S.	N.S.
Protein	*p* < 0.01 r = 0.178, *p* < 0.05	N.S.	N.S.
Ammonia	N.S.	N.S.	N.S.

N.S.: Not Significant, SPSS 30 The Mann–Whitney U test. Spearman’s rank correlation coefficient.

**Table 4:** Relationships between salivary biomarkers and oral function assessments.

		Cariogenic Bacteria	Acidity	Buffering Capacity	Leukocytes	Protein	Ammonia
Evaluation of the cariogenic bacterial count	Bacterial count	○					
Evaluation of the salivary flow rate	The Saxon test		○	○			
Evaluation of decreased occlusal force	Number of remaining teeth	○			○	○	
Evaluation of impaired tongue-lip motor function	/pa/				○		
	/ta/				○		
	/ka/				○		
Evaluation of masticatory dysfunction	Chewing ability	○			○	○	

○ The circles indicate correlations.

**Table 5:** Relationships between the salivary flow rate and oral function assessments.

			4. Tongue–Lip Motor Function					
	1. Oral Hygiene Status	3. Occlusal Force	/pa/	/ta/	/ka/	5. Masticatory Performance	6. Tongue Pressure	7. Swallowing Function
2. Salivary flow rate	N.S.	*p* < 0.01, r = 0.172, *p* < 0.03	N.S.	N.S.	N.S.	*p* < 0.01, r = 0.217, *p* < 0.01	N.S.	N.S.

N.S.: Not Significant. The Mann–Whitney U test, Spearman’s rank correlation coefficient, SPSS 30.

## Data Availability

The data presented in this study are available on request from the corresponding author. (The data are not publicly available due to privacy or ethical restrictions).
